# Vaccine therapies for glioma: clinical frontiers and potential breakthrough

**DOI:** 10.3389/fonc.2025.1613332

**Published:** 2025-06-25

**Authors:** Ying Xing, Caixia Liu, Yunfeng Feng, Shangyu Li, Yuping Chen

**Affiliations:** ^1^ Key Laboratory of Clinical Research on Respiratory and Digestive Diseases, Baoding Institute of Pediatrics, Baoding Hospital of Beijing Children’s Hospital, Capital Medical University, Baoding, Hebei, China; ^2^ Department of Research & Development, Celest Therapeutics (Shanghai) Co., Ltd, Shanghai, China

**Keywords:** glioma, vaccine, epitope spreading, neoantigen, antigen quality

## Abstract

Glioma, the most common primary malignant brain tumor, is characterized by high recurrence and mortality rates. Its effective treatment remains a therapeutic challenge in clinical neuro-oncology. Despite over twenty years of exploring new therapies for glioma, progress in improving patient survival outcomes has been limited. Tumor vaccines, as a promising therapeutic approach, may offer hope for glioma treatment. Currently, tumor vaccines targeting glioma include peptide vaccines, dendritic cell vaccines, and nucleic acid vaccines. Neoantigens, due to their high specificity and resistance to central immune tolerance, are ideal targets for tumor vaccines. Although promising results have been resulted in preclinical and clinical for glioma vaccines, there are still challenges impeding vaccine therapy for glioma. Therefore, future glioma vaccine applications must focus on the important roles of epitope spreading and antigen quality in enhancing immune response and therapeutic effectiveness. In this review, we discuss the current glioma vaccine antigen targets and types, introduce recent important clinical studies on glioma vaccines, and propose strategies to address potential barriers to vaccine application.

## 1 Introduction

Gliomas are a group of primary malignant tumors originating from glial cells or their precursor cells, accounting for 50% of central nervous system (CNS) malignant tumor (1). Gliomas typically exhibit locally invasive growth, with high heterogeneity and invasiveness, causing significant compression and destruction of surrounding brain tissues. Gliomas commonly occur between the ages of 21 and 50, with a peak incidence between 31 and 40 years old, though they are also relatively common in children around 10 years old (2, 3). According to the World Health Organization’s (WHO) grading system, gliomas are classified into four grades, with grades I and II being low-grade gliomas (LGG) and grades III and IV being high-grade gliomas (HGG). LGG usually grow slowly and have a good overall survival rate, while HGG grow rapidly with poor prognosis. For example, diffuse intrinsic pontine gliomas (DIPG) has a very poor prognosis, with a median survival time of approximately 9 to 12 months (4). Glioblastoma (GBM), is a high-grade (grade IV) glioma and is the most common malignant brain tumor in adults, accounting for about half of all gliomas. GBM has a high degree of malignancy, with a median survival time of only 15 months and a five-year survival rate of about 5.6% (5, 6).

The pathogenesis of gliomas involves highly complex mechanisms, with their biological basis rooted in the synergistic interplay of multi-level molecular pathological events (7). These tumors originate from neuroglial cells, driven primarily by genetic mutations and epigenetic dysregulation. Notably, IDH1/2 mutations induce abnormal accumulation of 2-hydroxyglutarate (2-HG), which disrupts epigenetic modifications and promotes metabolic reprogramming, serving as a hallmark of low-grade gliomas (8). Concurrently, TP53 mutations compromise genomic stability, while ATRX inactivation induces aberrant telomere maintenance mechanisms, collectively exacerbating tumor cell heterogeneity (9). In HGG, EGFR amplification (e.g., EGFRvIII mutation) and TERT promoter mutations cooperatively activate proliferative signaling and telomerase activity, fueling malignant progression (9). At the epigenetic level, MGMT promoter methylation compromises DNA repair mechanisms, thereby enhancing therapeutic resistance (10), whereas H3.K27M mutations disrupt chromatin architecture through abnormal histone modifications, a characteristic feature of pediatric diffuse midline gliomas (11). Dysregulation of key signaling pathways further amplifies malignancy: Overactivation of the RTK/RAS/PI3K pathway (e.g., EGFR/PDGFR aberrations) sustains proliferative and survival signals (12). Concomitant inactivation of the p53 pathway and defects in the RB pathway (e.g., CDKN2A/B deletion) synergistically drive cell cycle deregulation. Hypoxia-induced HIF-1α-mediated VEGF secretion orchestrates pathological angiogenesis (13). Within the tumor microenvironment, myeloid-derived suppressor cells (MDSCs) and regulatory T cells (Tregs) infiltration, coupled with PD-L1 overexpression, establish multi-layered immunosuppressive barriers. Meanwhile, matrix metalloproteinase (MMP)-mediated extracellular matrix remodeling facilitates tumor invasion (14). Metabolically, the Warburg effect underpins glycolytic reprogramming to fuel rapid proliferation (15). These interconnected mechanisms collectively manifest as hallmark clinical features of gliomas: infiltrative growth patterns, therapeutic resistance, and dismal prognosis.

Currently, the Stupp regimen remains the standard treatment for glioma, which involves maximal surgical resection of the tumor, followed by radiotherapy and chemotherapy (16). However, nearly all patients will experience recurrence after standard treatment, and tumor cells may develop resistance to chemotherapeutic drugs over the course of treatment, reducing therapeutic effectiveness (17). In recent years, numerous targeted inhibitors have entered clinical trials, yet their outcomes have been underwhelming. Bevacizumab, a vascular endothelial growth factor antibody, can extend progression-free survival but does not impact overall survival (18). Vorasidenib, an IDH inhibitor, has been shown to improve progression-free survival but is often associated with grade 3 or higher adverse events (19). Inhibitors targeting other pathways in gliomas, such as p53, retinoblastoma protein, and epidermal growth factor receptor amplification or mutations, have not improved patient prognosis in clinical trials (20). As an important component of tumor immunotherapy, therapeutic tumor vaccines have been proven effective against various solid tumors (21). In 2017, Ott et al. demonstrated that personalized neoantigens vaccines, designed using tumor genomic data of patients, could enhance their immune response and prolonging their overall survival time in melanoma patients (22). In 2019, Hilf et al. discovered that the APVAC1/2 vaccine showed robust immunogenicity in glioma patients, inducing specific immune responses and demonstrating good safety (23). These studies indicate that vaccine-based treatment strategies have revolutionized the treatment of various solid tumors.

Currently, more than 100 clinical trials for glioma vaccines are ongoing worldwide. Among them, the IDH1 short peptide vaccine (IDH1-vac) in Phase I clinical trials, while the dendritic cell (DC) vaccine DCVax-L in Phase III clinical trials, both showing efficacy (24, 25). This suggests that vaccine therapy holds significant potential in glioma treatment. This review briefly summarizes antigen classification and vaccine types for glioma, with a focus on clinical research related to glioma vaccines and the challenges and strategies involved in their application.

## 2 Glioma-related tumor antigens

**Figure 1:**
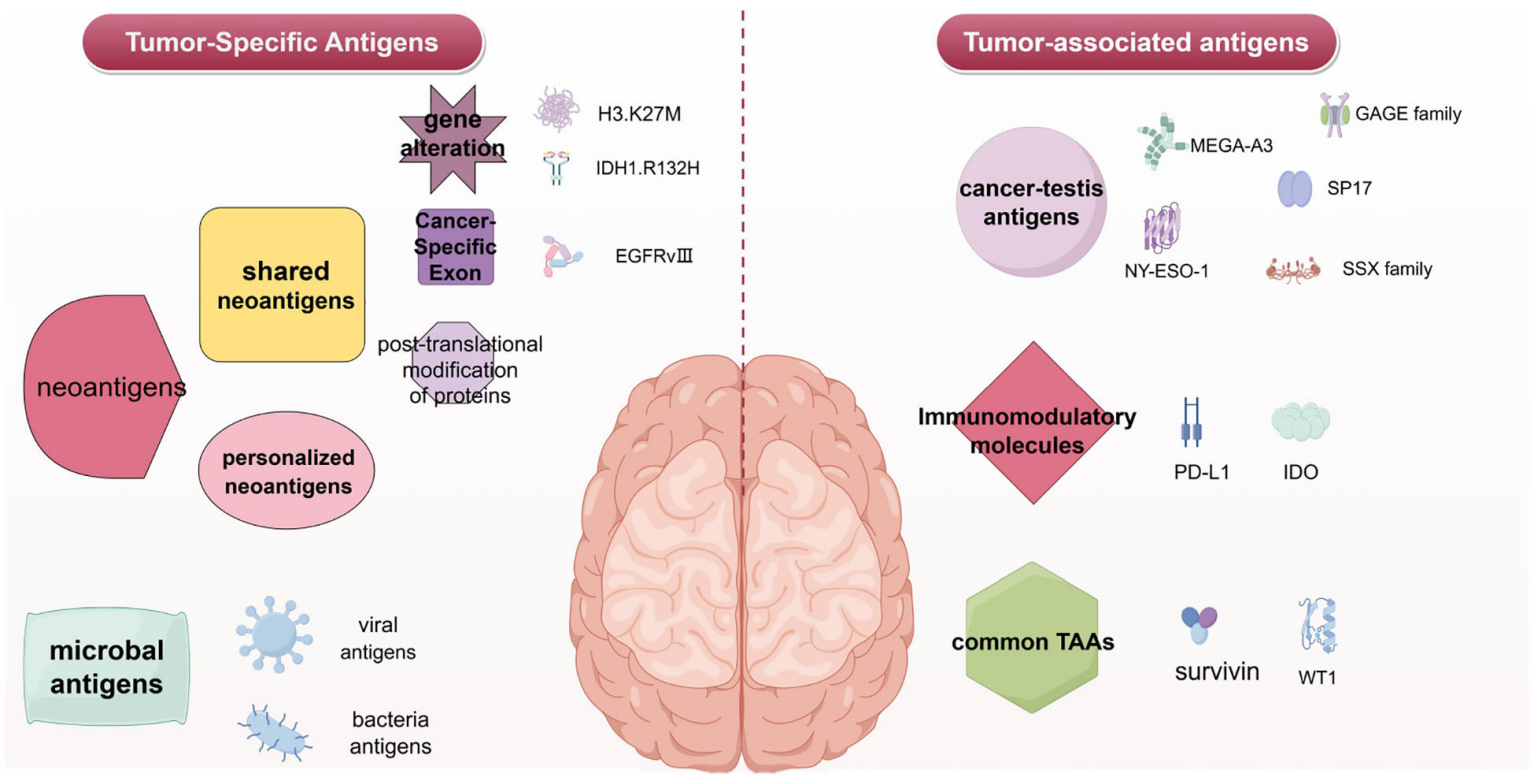
Glioblastoma vaccine antigen classification. Left, tumor-specific antigens for the Glioblastoma vaccine. Right, tumor-associated antigens for the Glioblastoma vaccine. The figure was generated by Figdraw (https://www.figdraw.com).

Tumor-associated antigens (TAAs) are a class of proteins expressed on the surface of tumor cells, which are also expressed at low levels in normal tissues. These antigens various origins, including abnormal differentiation of tumor cells, dysregulating of gene expression, and cellular stress responses (26, 27). The presence of TAAs in normal tissues leads to some degree of immune tolerance, making their immunogenicity relatively low and less likely to be effective targets for vaccines (28). However, the abnormal overexpression of TAAs in tumor cells or their aberrant expression in specific tissues provides an opportunity for the immune system to recognize and attack the tumor. Common TAAs that have shown potential in early - stage clinical trials include Survivin and WT1 (29, 30). Two special types of TAAs have greater potential as tumor vaccine targets: 1) Cancer-testis antigens (CTAs); 2) Immunoregulatory molecules. (**Figure 1**).

### 2.1 Cancer-testis antigens

Cancer-testis antigens (CTAs) are a subfamily of TAAs, known for their high tumor specificity. They have multiple biological functions, including promoting tumor cell proliferation, resistance to apoptosis, and facilitating tumor cells migration and invasion (31). Additionally, CTAs can induce both cellular and humoral immune responses, making them important targets for vaccines (32).

#### 2.1.1 MAGE-A3

MAGE-A3 is a CTA expressed in various tumors and has become a hot topic in vaccine research. MAGE-A3 belongs to the MAGE protein family, which is expressed during fetal development but is normally silenced in adult tissues. However, MAGE-A3 is aberrantly activated in several cancers, including melanoma, non-small cell lung cancer (NSCLC), bladder cancer, head and neck cancer, and glioma (33–37). The expression of MAGE-A3 in tumor cells enables the immune system to distinguish tumor cells from normal cells. Although its specific biological role in cancer progression has not been fully elucidated, MAGE-A3 is likely involved in regulating apoptosis and the cell cycle (38). Therapeutic cancer vaccines targeting MAGE-A3 are currently being developed and undergoing clinical trials to activate the patients’ immune systems to recognize and eliminate tumor cells expressing MAGE-A3 (39, 40). In melanoma and NSCLC, the immunotherapeutic strategies targeting MAGE-A3 have received particular attention (35, 41, 42). While early trials showed that the MAGE-A3 vaccine could induce immune responses and suggest potential survival benefits, Phase III trials in NSCLC failed to significantly improve disease-free survival in MAGE-A3-positive patients, leading to the termination of some studies (43).

#### 2.1.2 NY-ESO-1

NY-ESO-1, a member of the CTAs family, is recognized as one of the most immunogenic TAAs. Its ability to trigger both spontaneous humoral and cellular immune responses, coupled with its restricted expression in normal tissues, has established NY-ESO-1 as a leading target for tumor immunotherapy. Several clinical trials are currently underway to explore the therapeutic potential of NY-ESO-1-targeted tumor vaccines.

SCIB2 is a DNA vaccine encoding the NY-ESO-1 antigen and covering 16 NY-ESO-1 epitopes, which covers over 80% of HLA phenotypes (44). In murine B16-NY-ESO-1 tumor models, SCIB2 effectively inhibited the growth of tumor cells expressing, achieving a 35% long-term survival rate. Combinatorial regimens incorporating SCIB2 with Treg depletion, CTLA-4 blockade, or PD-1 blockade synergistically enhanced survival outcomes. The NY-ESO-1 DNA vaccine has completed Phase I clinical trial. This vaccine is safely administered via particle-mediated epidermal delivery (PMED) and was evaluated for safety and immunogenicity in patients who expressed the NY-ESO-1 antigen (45). Among 15 patients without antigen-specific immune responses prior to vaccination, 14 (93%) developed antigen-specific CD4^+^ T cell responses, and 5 (33%) exhibited CD8^+^ T cell responses. However, the persistence of the T cell response was not strong, potentially attributed to Treg-mediated immunosuppression. Temporal analysis revealed dynamic shifts in T cell epitope specificity, suggesting Treg-mediated modulation of effector T cell subsets may drive preferential suppression of distinct peptide-specific responses.

#### 2.1.3 Other CTAs

In addition to MAGE-A3 and NY-ESO-1, other CTA families such as the GAGE family, SP17, and SSX families are also considered important in relation to glioma (46, 47). Among these antigens, research has confirmed the potential of the Sp17 vaccine in a mouse ovarian cancer model. One Sp17 vaccine, which uses CpG as an adjuvant, effectively inhibits tumor growth and extends the OS in mice (48). Another vaccine, based on nanoparticle delivery of the hSp17_111–142_ peptide, induces a mixed Th1/Th2 response and further stimulates IgG1 and IgG2a production in B-cells, showing improved antibody immunogenicity with robust cross-reactivity (49). Collectively, these findings indicate that vaccines targeting specific CTAs have shown significant potential in tumor immunotherapy and may provide new strategies for treating malignant tumors like glioma. With the continuous advancements in immunotherapy technologies and clinical trials, the application prospects of these vaccines are expected to become clear.

### 2.2 Immunoregulatory molecules

Immunoregulatory molecules are immune - system inhibitory signaling pathways. Normally, co-stimulatory and co-inhibitory signals together regulate the immune response intensity to maintain immune tolerance. When tumors develop, they typically block immune- checkpoint signaling pathways to suppress immune responses, creating conditions for tumor growth and immune-surveillance evasion.

#### 2.2.1 PD-L1

Programmed cell death receptor-1 (PD-1) and its ligand, programmed cell death ligand-1 (PD-L1), belong to the cluster of differentiation 28 (CD28) and B7 families. They play an important role in T cell inhibition and exhaustion (50). Tumor cells exploit this mechanism by overexpressing PD-L1, thereby enabling immune escape and uncontrolled proliferation. The discovery of PD-1 and PD-L1 has revolutionized tumor immunotherapy. In 2016, the USC Norris Comprehensive Cancer Center developed a novel combination vaccine, PDL1-Vax, which integrates PD-1/PD-L1 immune checkpoint blockade with tumor-specific antigen recognition (51). This technique utilizes DCs loaded with PD-L1 antigen to stimulate anti-PD-L1 antibodies production, effectively counteracting tumor-mediated immunosuppression. By reactivating T cells to eliminate tumor cells and establishing long-term immunological memory in both T and B cells, PDL1-Vax offers broad-spectrum anticancer activity across cancer types. Given that over 70% of tumors escape immune surveillance via the PD-1/PD-L1 pathway, targeting PD-L1 can ensure the immune monitoring of PD-L1-high-expressing tumor cells, triggering their destruction upon dysregulation (52). This suggests that even if glioma cells or precancerous cells express PD-L1 to evade detection, they can be effectively blocked and destroyed by the PDL1-Vax vaccine, providing a new treatment strategy for glioma patients.

#### 2.2.2 IDO

Indoleamine 2,3-dioxygenase (IDO), a key enzyme in tryptophan metabolism, serves as a critical regulator of tumor immune evasion by suppressing T cell activity. IDO Overexpression has been observed in various tumors, including melanoma, prostate cancer, pancreatic cancer, and breast cancer (53–56). The upregulation of IDO drives tryptophan depletion and accumulation of downstream immunosuppressive metabolites, which can create an immunosuppressive microenvironment, fostering a tumor microenvironment that enables immune escape and resistance to cytotoxic immune responses (57). Preclinical studies indicate that an IDO-specific peptide vaccine (IDO vaccine) significantly inhibits tumor progression and prolongs survival in both IDO-secreting (B16F10 melanoma) and non-IDO-secreting (TC-1) murine models, highlighting its therapeutic potential regardless of baseline IDO expression (58). In December 2021, a phase I/II clinical study published in *Nature Medicine* reported that the IO Biotech dual-target vaccine (IO102/IO103) against IDO and PD-L1, combined with nivolumab, achieved a high objective response rate (ORR) of 80% and a median progression-free survival (PFS) of 26 months in metastatic melanoma (59). Which indicates that the vaccine-induced T cells can simultaneously recognize IDO/PD-L1-expressing tumor cells, reprogram the immunosuppressive tumor microenvironment towards an inflammatory state and enhance anti-tumor efficacy. Furthermore, at the 2024 European Society for Medical Oncology (ESMO) Congress, IO Biotech presented Phase II data showing that IO102-IO103 combined with pembrolizumab met its primary endpoint in advanced squamous cell carcinoma of the head and neck (SCCHN), with a median PFS of 6.6 months and a disease control rate of 66.7% (60). This evidence positions the IO102-IO103/anti-PD-1 combination as a promising first-line treatment for multiple cancers, including glioma, with favorable safety and efficacy profiles.

## 3 Tumor-specific antigens

Tumor-specific antigens (TSAs), uniquely expressed in tumor cells and absent in normal cells, trigger tumor-specific T cell response while minimizing off-tissue toxicity (61). TSAs arise from somatic mutations, generating neoantigens that have exhibit reduced central tolerance compared to self-epitopes, thereby enhancing the efficiency of tumor-targeted immune activation (62).

### 3.1 Neoantigens

Tumor neoantigens, generated from tumor-specific changes, exhibit higher tumor specificity and immunogenicity compared to TAAs, making them ideal for inducing tumor-specific immune responses. mRNA-lipid nanoparticle (LNP) vaccines targeting somatic mutation-derived neoantigens have demonstrated the capacity to generate long-lived CD8^+^ T cells in pancreatic ductal adenocarcinoma (PDAC) patients, overcoming a major obstacle in cancer vaccinology by producing long-lived and functional specific-T cells (63). Furthermore, neoantigen diversity originates from multiple mechanisms, including somatic genomic variations, transcriptional abnormalities, post-translational modifications, and viral open reading frames (ORFs) (64, 65).

#### 3.1.1 Shared tumor neoantigens

Shared tumor neoantigens are immunogenic epitopes commonly presented in tumor patients, typically derived from hotspot mutations in tumor-driving genes. These neoantigens are selectively expressed in tumor tissues and serve as tumor-specific antigens that trigger immune responses (66). Shared tumor neoantigens have become a focal point in cancer vaccine due to their unique advantages, including “off-the-shelf” availability, general applicability, high tumor specificity and robust immunogenicity (67).

##### 3.1.1.1 Cancer-specific exons

Cancer-specific exons (CSEs) are exon sequences exclusively expressed in tumor, with absent or minimal expression in normal cells. Some CSEs may originate from tumor-specific alternative splicing or other oncogenic mechanisms. Proteins encoded by CSEs are often localized to the cell surface or extracellular matrix, endowing them with high tumor specificity and immunogenic visibility. In 2024, researchers at St. Jude Children’s Research Hospital employed computational biology tools to analyze 1532 RNA sequencing datasets spanning 16 types of pediatric solid tumors and gliomas, identifying 2933 CSEs involving 157 genes (68). A study published in *Nature* demonstrated that dysregulated RNA splicing in tumors generates immunogenic neoantigens. Through integrative analysis of > 5000 samples spanning 12 cancer types, researchers identified recurrent splicing errors in genes such as GNAS and RPL22 that exhibit cross-tumor stability and are effectively recognized by adaptive immunity (69). In *in vitro* functional validation revealed that engineered T cells therapies eliminated 80% of cancer cells carrying GNAS splice variants within 72 hours, establishing a mechanistic for pan-cancer vaccine development. The high tumor specificity of CSE-encoded proteins positions them as compelling immunotherapeutic targets. For example, the common EGFRvIII mutation found in adult glioma is a type of CSEs. EGFRvIII, a mutant form of the epidermal growth factor receptor (EGFR), is commonly expressed in glioma and prevalent in ~50% of GBM cases (70, 71). Due to a deletion in its domain, EGFRvIII exhibits distinct properties compared to the wild-type EGFR, including stable expression on the tumor cell surface and continuous signaling activity, making it a popular target for vaccine development. Rindopepimut (CDX-110) is a peptide vaccine could induce EGFRvIII-specific immune responses (72). Phase II trials reported prolonged progression-free survival and overall survival (OS) in vaccinated patients. However, the subsequent international Phase III ACT IV trial failed to meet its primary endpoint, with median the OS of 20.4 months 20.4 months (rindopepimut) versus 21.1 months (control) (73). This discrepancy may stem from chemotherapy-induced immunosuppression, insufficient EGFRvIII-specific T cell priming, or antigen loss via tumor heterogeneity-driven immune evasion.

##### 3.1.1.2 IDH1.R132H

Isocitrate dehydrogenase type 1 (IDH1) serve as specific molecular markers in gliomas. Since the initial discovery of IDH1 driver mutations in glioma by Professor Parsons DW in 2008, the role and clinical significance of these mutations have gradually become a major focus in glioma research (74). These mutations induce abnormal accumulation of the metabolite 2-hydroxyglutarate (2-HG), which disrupts cellular epigenetic modifications and promotes tumorigenesis. As a tumor driver, IDH1 mutations demonstrate particular promise as immunotherapeutic targets for vaccine development. Studies revealed that IDH1 mutations occur in approximately 80-90% of LGGs, including astrocytomas and oligodendrogliomas. The most frequent IDH1 mutation in gliomas occurs at codon 132, resulting in an arginine-to-histidine substitution IDH1(R132H) (75–77). The IDH1(R132H) mutation is recognized as a potential neoantigen in WHO Grade II and III diffuse astrocytomas. This mutant protein can be specifically recognized by the immune system, eliciting mutation-specific CD4^+^ T-helper 1 (Th1) response (78). Platten et al. designed a series of peptides encompassing the R132H mutation and identified peptide p123-142, which spans codons 123–142 and includes the R132H substitution, as a potent inducer of antitumor immunity against IDH1-mutanted cells (79). Preclinical studies utilizing MHC-humanized mouse models have shown that the IDH1(R132H)-specific peptide vaccine (IDH1-vac) can induce sustained antitumor-specific T helper cell responses (80, 81). Based on these preclinical findings, researchers conducted a multicenter phase I clinical trial to evaluated the safety, feasibility, and immunotherapeutic efficacy of mutant IDH1-targeted vaccination in newly diagnosed WHO grade III and grade IV glioma patients (24). The trial outcomes revealed that IDH1-vac effectively primed tumor-reactive T cell responses in HGG patients compared to control groups. Importantly, the vaccine demonstrated favorable safety profiles and robust immunogenicity, with evidence of improved clinical outcomes in IDH1-mutant astrocytoma cohorts. These findings provide critical validation for further development of mutation-specific vaccine strategies in neuro-oncology.

##### 3.1.1.3 H3.K27M

In 2012, exome sequencing of pediatric gliomas and whole-genome analysis of DIPGs led to the identification of somatic H3.K27M mutations (82, 83). Genomic characterization studies subsequently established that these H3.K27M mutations may occur in either histone H3 variants H3.1 (HIST1H3B/C) or H3.3 (H3F3A), functioning as early driver events that are clonally maintained throughout tumor evolution (84, 85). The molecular hallmark of H3.K27M-mutant gliomas involves global reduction of H3.K27 trimethylation (H3.K27me3) coupled with increased H3.K27 acetylation (H3.K27ac). This epigenetic reprogramming creates an oncogenic chromatin state characterized by polycomb repressive complex 2 (PRC2) dysfunction, leading to tumor suppressor gene silencing and proto-oncogenes activation. Epidemiologically, H3.K27M mutations are detected in ~80% of pediatric DIPG cases (86), while adults with diffuse midline gliomas (DMGs) show H3.K27M mutation frequencies ranging from 15% to 60% across exhibit different cohorts (87–89). The 2021 WHO Classification of Central Nervous System Tumors formally recognized the diagnostic significance of this alteration, subsequently classifying H3.K27M-mutated DMGs as a distinct molecular entity within the grade IV glioma category.

Through MHC-binding affinity prediction algorithms, a synthesized H3.K27M peptide (H3.3K27M 26-35) was identified as a high-affinity epitope for HLA-A*0201, a prevalent human leukocyte antigen subtype (90). Preclinical validation in murine models demonstrated that this peptide induced mutation-specific immune responses, including cytotoxic CD8^+^ T cell responses and Th1-polarized CD4^+^ T cell activation (90). Notably, adoptive transfer of TCR-engineered CD8+ T cells targeting this epitope significantly prolonged survival in glioma xenograft model (91). A Phase I/II clinical trial of the H3.K27M synthetic peptide vaccine is currently underway to evaluate its feasibility and efficacy in newly diagnosed pediatric patients with H3.K27M-mutant DIPG or non-bridging DMG. As the vaccine targets human leukocyte antigen (HLA)-restricted epitopes, eligibility is limited to HLA-A2 (02:01)-positive patients. Results from this study have shown that 6 of 18 DIPG patients developed robust immune response against the H3.K27M peptide (92). In a separate recent study evaluating the safety, tolerability, and immunogenicity of long peptide vaccines targeting H3.K27M mutations in adult progressive H3.K27M^+^ DMG, five of eight patients exhibited dominant mutation-specific immune response mediated primarily by CD4^+^ T cells. Notably, one patient exhibited a potent mutation-specific T cell response and achieved sustained complete remission lasting over 31 months (93).

#### 3.1.2 Personalized neoantigens

Personalized neoantigens are immunogenic mutations uniquely expressed in individual patients. These neoantigens are absent in normal tissues and are not subject to central or peripheral immune tolerance mechanisms. By engaging MHC molecules to activate CD4^+^ T cells and CD8^+^ T cells, personalized neoantigens elicit tumor-specific immune responses that suppress tumor growth (62).

Identifying personalized neoantigens is a critical step toward achieving individualized therapy for glioma patients. Currently, two primary methodologies are employed for personalized neoantigens identification: genomic and proteomic approaches. The genomic approach entails the use of high-throughput sequencing technologies to analyze tumor biopsies and matched non-tumor tissue samples (typically peripheral blood mononuclear cells, PBMCs) from patients. DNA and RNA sequencing data are integrated to predict potential neoantigenic mutations through bioinformatics algorithms (94, 95). Following genomic analysis, bioinformatics algorithms are employed to predict potential personalized neoantigen peptides with predicted binding affinity to MHC molecules (96, 97). In contrast, the proteomic approach utilizes mass spectrometry to isolate HLA-peptide complexes from tumor tissue via affinity chromatography. Subsequent sequencing and comparison of peptide sequences with the patient’s germline genome enables the identification of tumor-specific antigenic peptides presented on the cells surface.

However, not all somatic mutations generate immunogenic epitopes recognizable by the immune system, primarily due to HLA restriction. When predicting potential immunogenic epitopes, HLA haplotypes must be incorporated into the computational pipeline. Peptides demonstrating high binding affinity to MHC-I molecules (defined as IC50 <150 nmol/l) are prioritized as candidates for eliciting CD8^+^ T cell response (98). To systematically predict HLA-presented epitopes, multiple bioinformatics tools have been developed, including NetMHCpan, MHCflurry and DeepHLApan (99–101). Among these, NetMHCpan enhances neoantigen prediction accuracy by integrating both MHC-peptide binding affinity data and mass spectrometry (MS)-validated peptide elution profiles, thereby enabling pan-specific coverage of MHC-I alleles (101). While most prediction algorithms focus on MHC-I epitopes, the inherent structural flexibility of MHC-II peptide-binding grooves poses significant challenges for computational modeling. Emerging tools such as MixMHC2pred, NetMHCIIpan, and neonmhc2 aim to address this gap (102–104). Nevertheless, current prediction accuracy for MHC class II-peptide interactions remains inferior to that of MHC class I systems. Nevertheless, current prediction accuracy for MHC-II-peptide interactions remains inferior to that of MHC-I systems.

Personalized neoantigen-targeting tumor vaccines have demonstrated preliminary clinical efficacy in recent trials. Keskin et al. treated eight glioma patients with a multi-epitope personalized neoantigen vaccine (105). Immunological analysis revealed that patients who abstained from dexamethasone during the vaccination period developed sustained circulating neoantigen-specific CD4^+^ and CD8^+^ T cell responses characterized by: enhanced memory T cell phenotypes, increased tumor-infiltrating lymphocytes (TILs) densities, and vaccine-induced T cell trafficking from peripheral circulation to intracranial tumor sites, collectively resulting in immunomodulation of glioma microenvironment. Complementing these findings, a collaborative study from Yale University and Dana-Farber Cancer Institute, published in *Nature*, evaluated a personalized neoantigen vaccine in 9 surgically resected high-risk renal cell carcinoma patients (106). The intervention elicited potent personalized neoantigen-specific T cell clonal expansion, with all patients remaining recurrence-free at a median follow-up time of 40.2 months.

### 3.2 Microbiological antigens

Microbial antigens are typically defined as pathogen-derived molecular components (e.g., viral proteins or bacterial surface molecules) that persist following acute infections. When such infections remain unresolved, latent pathogens may establish chronic persistence within host cells, potentially driving oncogenic transformation through sustained inflammatory signaling or direct genomic damage. Consequently, harnessing pathogen-specific T cell immunity against these microbial antigens has emerged as a strategic approach to induce cross-reactive immune responses targeting tumor cells.

#### 3.2.1 Viral antigens

Viral antigens are immunogenic proteins encoded by viral genomes that are aberrantly expressed in tumor cells and recognized as non-self by the immune system. These antigens typically originate from conserved epitopes within viral structural or oncogenic proteins, which are processed and presented via MHC molecules on the surface of transformed cells, thereby marking them for immune-mediated destruction. Notably, oncogenic viruses such as human papillomavirus (HPV), hepatitis B virus (HBV), Epstein-Barr virus (EBV), and human T-lymphotropic virus 1 (HTLV-1) are etiologically linked to malignancies through persistent expression of their viral antigens. Currently therapeutic strategies focus on developing vaccines targeting these cancer-associated viral antigens, with several candidates in active clinical evaluation. For example, Preventive vaccines including Gardasil and Cervarix, which target HPV types 16 and 18, have demonstrated remarkable efficacy in reducing cervical cancer incidence through pre-exposure immunization (107).

Cytomegalovirus (CMV) phosphorylated protein 65 (pp65), a dominant immunogenic antigen encoded by the CMV genome, plays a critical role in viral pathogenesis. While CMV establishes lifelong latency in immunocompetent hosts with asymptomatic persistence, viral reactivation has been epidemiologically associated with oncogenesis, particularly in gliomas and melanoma. Molecular analyses reveal CMV pp65 gene expression in 86-90% of glioma specimens (108, 109), 69.7% of melanoma tumors (110), and > 90% of breast carcinomas (111). The therapeutic potential of CMV pp65-targeted vaccines has been most extensively explored in glioma, particularly GBM. VBI-1901 is a CMV pp65-based vaccine that received FDA Fast Track designation in June 2021 for treating recurrent GBM following first relapse. The Phase I/II multicenter, randomized, open-label trial of VBI-1901, presented at the 2024 World Vaccine Congress, demonstrated preliminary clinical efficacy in a multicenter, randomized, controlled, open-label study recurrent GBM. Among 16 evaluable patients, the study achieved a disease control rate (DCR) of 44%, with one exceptional responder maintaining survival beyond 40 months as of August 2023 follow-up (ClinicalTrials.gov Identifier: NCT03382977). These findings indicate the therapeutic potential of CMV pp65-targeted immunotherapy in glioma treatment.

#### 3.2.2 Bacterial antigen

Recent studies revealed that intratumoral bacteria, prevalent in multiple solid malignancies, critically influence oncogenesis and metastasis progression (112, 113). These commensal microbes elicit MHC-I restricted T cell responses through molecular mimicry between bacterial antigens and tumor-associated epitopes, suggesting cross-reactive immunosurveillance against malignant cells. In immunosuppressive microenvironments, intratumoral bacteria can be targeted with antibiotics, leading to the release of microbial antigens. Which effectively convert immunologically “cold” tumors into “hot” phenotypes, enabling the immune system to recognize and attack both infected and uninfected tumor cells (114). Researchers analyzed17 melanoma specimens and found 41 types of bacteria. HLA-peptidomics analysis revealed 248 bacterially derived peptides presented on tumor cell surfaces (115). Current research priorities include systematic identification of immunodominant bacterial epitopes through multi-omics integration to accelerate microbial antigen-targeted vaccine design. Functional validation demonstrated T cell receptor-mediated recognition of these bacterial peptides, positioning them as non-self neoantigens for vaccine development. Complementing these findings, a study published in *Science Translational Medicine* engineered tumor-homing attenuated Listeria monocytogenes expressing tetanus toxoid (TT) in PDAC models (116). Pre-immunization against TT enabled recall CD4^+^ T cell responses targeting tumor cells presenting TT epitopes, achieving significant intratumoral lymphocyte infiltration. Current researches priorities include systematic identification of immunodominant bacterial peptides through multi-omics integration to accelerate the development of bacterial antigen-targeted vaccines.

## 4 Vaccine type

**Figure 2:**
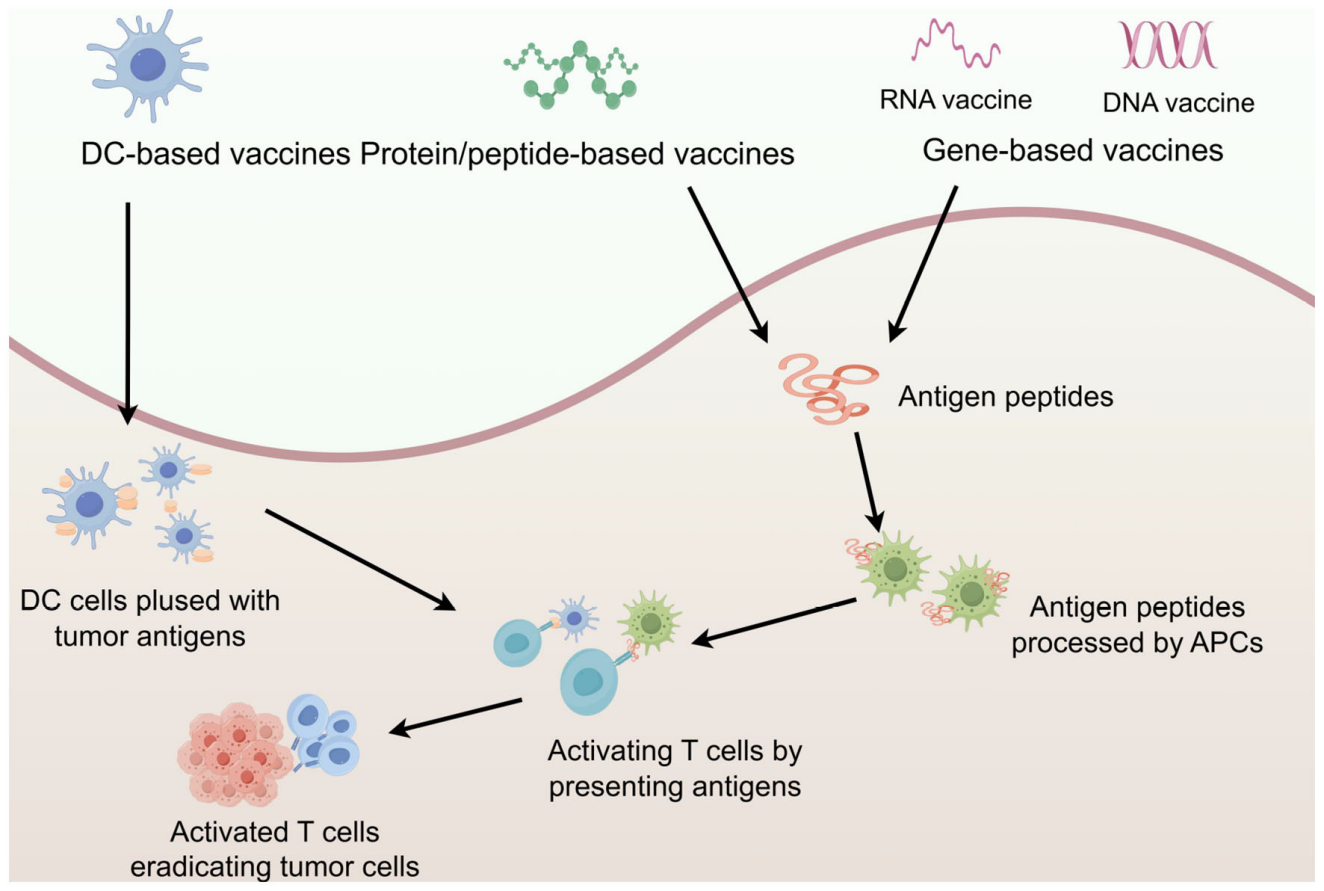
Major types of vaccine. *In vivo*, antigens are eventually presented to T cells to induce specific immune responses and achieve anti-tumor effects. The figure was generated by Figdraw (https://www.figdraw.com).

Tumor vaccines are biologics designed to stimulate or restore the immune system’s ability to recognize and destroy cancer cells. These vaccines contain fragments of tumor-specific antigens (TSAs) or tumor-associated antigens (TAAs) and adjuvant active ingredients. After being taken up and processed by antigen-presenting cells (APCs), these antigens activate and expand tumor-specific T cells, thereby enhancing the body’s resistance to tumors. Tumor vaccines are primarily used as adjuvant therapy following radiotherapy, chemotherapy, or surgical resection. For instance, dendritic cell (DC) vaccines are one type of tumor vaccine. Therapeutic tumor vaccines can be categorized into three main types based on the source of the tumor antigen expressed: protein/peptide vaccines, DC vaccines, and nucleic acid vaccines (including DNA and RNA vaccines) (**Figure 2**).

### 4.1 Protein/peptide vaccines

Protein/peptide vaccines induce tumor-specific immune responses by activating T cells targeting tumor-associated epitopes. These synthetic antigens are internalized by DCs, processed through the MHC-I and MHC-II antigen presentation pathways, and presented on the cell surface to prime cytotoxic CD8^+^ T cells and CD4^+^ helper T cells. While MHC-I-restricted short peptides (8–10 amino acids) are commonly used, their clinical utility is limited by low immunogenicity and off-target binding to non-professional antigen-presenting cells (117, 118). Consequently, research has shifted toward long peptide vaccines (typically 15–30 amino acids), which preferentially traffic to professional antigen-presenting cells (APCs). Following endocytosis, long peptides undergo partial proteolytic cleavage in endosomal compartments. Processed epitopes are then either: Loaded onto MHC-II molecules to activate CD4^+^ T-helper cells, or cross-presented via MHC-I (119). This dual activation mechanism enables long peptide vaccines to induce polyfunctional T cell responses with enhanced durability. In a Phase II trial, IMA-950, a multi-peptide vaccine containing 11 tumor-associated peptides for glioma, showed a 30% immune response rate in newly diagnosed HLA-A2-positive patients. Moreover, patients developing injection site reactions demonstrated a significantly prolonged mOS compared to non-responders. However, combining IMA-950 with the TLR3 agonist poly-ICLC failed to improve PFS or OS in HGG patients cohorts, highlighting the need for optimized adjuvant strategies (120).

### 4.2 Dendritic cells vaccine

DCs, as the most potent APCs, serve as critical bridges between innate and adaptive immune. By capturing and presenting tumor antigens through multiple mechanisms, DCs elicit antigen-specific anti-tumor immune responses. Capitalizing on this characteristic, DC vaccine therapy has been developed through *ex vivo* activation of autologous DCs followed by reinfusion to activate cytotoxic T cells against tumor cells (121). Clinical investigations of DC vaccines in glioma demonstrated progressive success. A 2021 Phase I trial evaluating neoantigen DC vaccine (Neo-DCVac) in 12 patients with advanced metastatic lung cancer reported a mOS of 7.9 months with favorable safety (122). Combination therapy with immune checkpoint inhibitors (ICIs) revealed synergistic efficacy through enhanced T cell responses (123). The landmark DCVax-L trial, utilizing patient-derived DCs loaded with autologous tumor antigens, demonstrated significant survival benefits in a Phase III controlled study (25). When combined with standard treatment (SOC), this personalized immunotherapy achieved unprecedented outcomes - becoming the first intervention in two decades to improve survival in newly glioma patients, and the first in three decades to show significant survival extension in recurrent glioma, while maintaining an excellent safety profile.

### 4.3 Nucleic acid vaccines

Nucleic acid vaccines (encompassing DNA and RNA vaccines) deliver nucleotide sequences encoding TSAs into host cells, enabling endogenous antigen expression to elicit anti-tumor immunity (124).

DNA vaccines exhibit distinct advantages including thermal stability, rapid design and production scalability. However, their clinical application faces challenges such as potential genetic integration risks and suboptimal immunogenicity, necessitating co-administration with adjuvants or combinatorial immunotherapy (125). Preclinical studies demonstrated that neoantigen-targeted DNA vaccines predominantly activate MHC I-restricted CD8^+^ T cell responses, generating potent anti-tumor immunity (126, 127). For example, a lipid-nanoparticle-encapsulated multi-epitope DNA vaccine in B16 melanoma murine models induced tumor-infiltrating CD8^+^ T cells, achieving significant suppression of melanoma growth and reduction of pulmonary metastatic nodules (128).

In contrast to DNA vaccines, mRNA-based vaccines demonstrate enhanced immunogenicity, superior design flexibility, shorter production times, and unique cytoplasmic translation mechanism that eliminates genomic integration risks. Despite being more prone to degradation than DNA, their stability can be enhanced through lipid nanoparticles or stabilizing adjuvant formulations. A breakthrough mRNA vaccine engineered at the University of Florida employs a “onion-like” multi-layer RNA lipid nanoparticle aggregation system (RNA-LPA) to deliver patient-derived tumor-associated mRNA, rapidly activating the immune system and remodeling of the immunosuppressive tumor microenvironment (TME) (129). Autogene cevumeran is a personalized neoantigen vaccine utilizing optimized uridine mRNA-lipid nanoparticles, engineered to induce long-lived, functional CD8^+^ T cells that persist for years while maintaining effector functions in in patients with PDAC (63). In a phase Ib clinical trial (NCT04161755), autogene cevumeran demonstrated potential to delay disease recurrence, with vaccinated patients exhibiting significantly longer median recurrence-free survival compared to unvaccinated controls (15.4 vs 8.8 months; HR 0.42).

## 5 Clinical research status of glioma vaccines

Therapeutic cancer vaccines have evolved through decades of preclinical and clinical optimization, marking a revolution in immuno-oncology. Neoantigen-based strategies now spearhead the development of personalized immunotherapies, leveraging patient-specific immune cells to mediate tumor-specific cytotoxicity through MHC-restricted mechanisms. In glioma therapeutics, multiple vaccine approaches, including peptide vaccines, DC vaccines, and mRNA vaccines, have demonstrated clinically meaningful outcomes (**Table 1**). As of September 30, 2024, a total of 164 clinical trials related to glioma vaccines have been registered on the international clinical trials platform (http://www.clinicaltrials.gov) under the search strategy: condition = “glioma” AND intervention = “vaccine”.

**Table 1:** Clinical trials of glioma therapeutic vaccines.

Identifier	Title	Vaccine type	Phase	Treatment approach	Conditions	Status
NCT03149003	A Study of DSP-7888 Dosing Emulsion in Combination With Bevacizumab in Patients With Recurrent or Progressive Glioblastoma Following Initial Therapy	Peptide Vaccine	PHASE3	DSP-7888 Dosing Emulsion Bevacizumab	Glioblastoma	COMPLETED
NCT05096481	PEP-CMV Vaccine Targeting CMV Antigen to Treat Newly Diagnosed Pediatric HGG and DIPG and Recurrent Medulloblastoma	Peptide Vaccine	PHASE2	PEP-CMV Temozolomide Tetanus Diphtheria Vaccine	High Grade Glioma Diffuse Intrinsic Pontine Glioma Recurrent Medulloblastoma	RECRUITING
NCT02358187	A Vaccine Trial for Low Grade Gliomas	Peptide Vaccine	PHASE2	HLA-A2 Restricted Glioma Antigen-Peptides with Poly-ICLC	Low Grade Glioma	RECRUITING
NCT02754362	A Toll-like Receptor Agonist as an Adjuvant to Tumor Associated Antigens (TAA) Mixed With Montanide ISA-51 VG With Bevacizumab for Patients With Recurrent Glioblastoma	Peptide Vaccine	PHASE2	Bevacizumab Peptide Vaccine Poly-ICLC as immune adjuvant| Keyhole limpet hemocyanin (KLH)	Glioblastoma Glioma	WITHDRAWN
NCT00643097	Vaccine Therapy in Treating Patients With Newly Diagnosed Glioblastoma Multiforme	Peptide Vaccine	PHASE2	PEP-3 vaccine sargramostim Temozolomide	Malignant Neoplasms of Brain	COMPLETED
NCT02455557	SurVaxM Vaccine Therapy and Temozolomide in Treating Patients With Newly Diagnosed Glioblastoma	Peptide Vaccine	PHASE2	Laboratory Biomarker Analysis Montanide ISA 51 VG Sargramostim SVN53-67/M57-KLH Peptide Vaccine Temozolomide	Glioblastoma Gliosarcoma	ACTIVE_NOT_RECRUITING
NCT04280848	Anticancer Therapeutic Vaccination Using Telomerase-derived Universal Cancer Peptides in Glioblastoma	Peptide Vaccine	PHASE2	UCPVax Temozolomide	Glioblastoma	ACTIVE_NOT_RECRUITING
NCT05163080	SurVaxM Plus Adjuvant Temozolomide for Newly Diagnosed Glioblastoma (SURVIVE)	Peptide Vaccine	PHASE2	SurVaxM	Newly Diagnosed Glioblastoma	ACTIVE_NOT_RECRUITING
NCT01920191	Phase I/II Trial of IMA950 Multi-peptide Vaccine Plus Poly-ICLC in Glioblastoma	Peptide Vaccine	PHASE1|PHASE2	IMA 950	Acute Myeloid Leukemia Myelodysplastic Syndromes Glioblastoma Multiforme Melanoma Non-Small Cell Lung Cancer Ovarian Cancer Pancreatic Cancer Sarcoma Renal Cell Carcinoma	COMPLETED
NCT02454634	Phase I Trial of IDH1 Peptide Vaccine in IDH1R132H-mutated Grade III-IV Gliomas	Peptide Vaccine	PHASE1	DRUG: IDH1 peptide vaccine	Malignant Glioma	COMPLETED
NCT02960230	H3.3K27M Peptide Vaccine With Nivolumab for Children With Newly Diagnosed DIPG and Other Gliomas	Peptide Vaccine	PHASE1|PHASE2	K27M peptide DRUG: Nivolumab	Glioma Malignant Glioma Astrocytoma Grade II Oligodendroglioma Glioma Astrocytic Oligoastrocytoma	COMPLETED
NCT00795457	Effects of Vaccinations With HLA-A2-Restricted Glioma Antigen-Peptides in Combination With Poly-ICLC for Adults With High-Risk WHO Grade II Astrocytomas and Oligo-Astrocytomas	Peptide Vaccine	EARLY_PHASE1	GAA/TT-peptide vaccine and poly-ICLC	Malignant Glioma Astrocytoma Glioblastoma	COMPLETED
NCT00874861	HLA-A2-Restricted Glioma Antigen-Peptides Vaccinations With Poly-ICLC for Recurrent WHO Grade II Gliomas	Peptide Vaccine	EARLY_PHASE1	Peptide vaccine + Poly-ICLC	Brain Cancer Brain Neoplasm Primary Brain Neoplasms Recurrent Brain Tumor Cancer of the Brain	COMPLETED
NCT02193347	IDH1 Peptide Vaccine for Recurrent Grade II Glioma	Peptide Vaccine	PHASE1	PEPIDH1M vaccine Tetanus-Diphtheria Toxoid (Td) DRUG: Temozolomide	Brain and Central Nervous System Tumors	COMPLETED
NCT01522820	Vaccine Therapy, Temozolomide, and Radiation Therapy in Treating Patients With Newly Diagnosed Glioblastoma Multiforme	Peptide Vaccine	PHASE1	glioblastoma multiforme multipeptide vaccine IMA950	Malignant Neoplasms Brain	COMPLETED
NCT01403285	Peptide-based Glioma Vaccine IMA950 in Patients With Glioblastoma	Peptide Vaccine	PHASE1	Cyclophosphamide IMA950 plus GM-CSF	Brain and Central Nervous System Tumors	TERMINATED
NCT00293423	GP96 Heat Shock Protein-Peptide Complex Vaccine in Treating Patients With Recurrent or Progressive Glioma	Peptide Vaccine	PHASE1|PHASE2	BIOLOGICAL: HSPPC-96|PROCEDURE: Standard Surgical Resection	Glioblastoma Multiforme	COMPLETED
NCT00576537	Tumor Lysate Pulsed Dendritic Cell Immunotherapy for Patients With Brain Tumors	DC Vaccine	PHASE2	Dendritic Cell Immunotherapy	Giant Cell Glioblastoma Glioblastoma Gliosarcoma	COMPLETED
NCT01006044	Efficacy & Safety of Autologous Dendritic Cell Vaccination in Glioblastoma Multiforme After Complete Surgical Resection	DC Vaccine	PHASE2	autologous dendritic cells	Brain and Central Nervous System Tumors Gastrointestinal Stromal Tumor Sarcoma	COMPLETED
NCT04277221	ADCTA for Adjuvant Immunotherapy in Standard Treatment of Recurrent Glioblastoma Multiforme (GBM)	DC Vaccine	PHASE3	Autologous Dendritic Cell/Tumor Antigen, ADCTA	Glioblastoma Multiforme	UNKNOWN
NCT05100641	AV-GBM-1 vs Control as Adjunctive Therapy Following Surgery and RT/TMZ in Newly Diagnosed GBM	DC Vaccine	PHASE3	AV-GBM-1 Autologous monocytes	Primary Glioblastoma	NOT_YET_RECRUITING
NCT00045968	Study of a Drug [DCVax庐-L] to Treat Newly Diagnosed GBM Brain Cancer	DC Vaccine	PHASE3	Dendritic cell immunotherapy	Glioblastoma Multiforme Glioblastoma GBM Grade IV Astrocytoma	UNKNOWN
NCT01759810	Proteome-based Personalized Immunotherapy of Glioblastoma	DC Vaccine	PHASE2|PHASE3	Dendritic vaccine allogeneic hematopoietic stem cells, cytotoxic lymphocytes Dendritic vaccine, autologous hematopoietic stem cell	Glioblastoma	UNKNOWN
NCT02465268	Vaccine Therapy for the Treatment of Newly Diagnosed Glioblastoma Multiforme	DC Vaccine	PHASE2	pp65-shLAMP DC with GM-CSF unpulsed PBMC and saline Td, Saline pp65-flLAMP DC with GM-CSF	Glioblastoma Multiforme Glioblastoma,Malignant Glioma Astrocytoma, Grade IV	COMPLETED
NCT01567202	Study of DC Vaccination Against Glioblastoma	DC Vaccine	PHASE2	Surgery Chemotherapy Radiotherapy DC vaccination	Glioma Glioblastoma Multiforme Neoplasms	UNKNOWN
NCT01204684	Dendritic Cell Vaccine for Patients With Brain Tumors	DC Vaccine	PHASE2	autologous tumor lysate-pulsed DC vaccination Tumor lysate-pulsed DC vaccination+0.2% resiquimod Tumor-lysate pulsed DC vaccination +adjuvant polyICLC	Glioma|Anaplastic Astrocytoma Anaplastic Astro-oligodendroglioma|Glioblastoma	COMPLETED
NCT01213407	Dendritic Cell Cancer Vaccine for High-grade Glioma	DC Vaccine	PHASE2	Trivax, Temozolomide, Surgery, Radiotherapy Temozolomide, Surgery, Radiotherapy	Glioblastoma Multiforme	COMPLETED
NCT02772094	Dendritic Cell-Based Tumor Vaccine Adjuvant Immunotherapy of Human Glioblastoma Multiforme (WHO Grade IV Gliomas)	DC Vaccine	PHASE2	Single arm, open-label	Glioblastoma Multiforme Glioblastoma	UNKNOWN
NCT04523688	Vaccination with Autologous Dendritic Cells Loaded with Autologous Tumour Homogenate in Glioblastoma	DC Vaccine	PHASE2	Autologous Dendritic Cells (DC) vaccine Temozolomide	Glioblastoma Vaccination	RECRUITING
NCT04888611	Neoadjuvant PD-1 Antibody Alone or Combined With DC Vaccines for Recurrent Glioblastoma	DC Vaccine	PHASE2	Camrelizumab plus GSC-DCV Camrelizumab plus Placebo	Recurrent Glioblastoma	UNKNOWN
NCT01006044	Efficacy & Safety of Autologous Dendritic Cell Vaccination in Glioblastoma Multiforme After Complete Surgical Resection	DC Vaccine	PHASE2	autologous dendritic cells	Glioblastoma Multiforme	COMPLETED
NCT03014804	Autologous Dendritic Cells Pulsed With Tumor Lysate Antigen Vaccine and Nivolumab in Treating Patients With Recurrent Glioblastoma	DC Vaccine	PHASE2	autologous dendritic cells pulsed with tumor lysate antigen Vaccine Laboratory Biomarker Analysis Nivolumab Quality-of-Life Assessment Questionnaire Administration	Giant Cell Glioblastoma Oligodendroglioma Recurrent Glioblastoma Small Cell Glioblastoma	WITHDRAWN
NCT01957956	Vaccine Therapy and Temozolomide in Treating Patients With Newly Diagnosed Glioblastoma	DC Vaccine	EARLY_PHASE1	Malignant Glioma Tumor Lysate-Pulsed Autologous Dendritic Cell Vaccine	Glioblastoma Multiforme	COMPLETED
NCT00323115	Phase II Feasibility Study of Dendritic Cell Vaccination for Newly Diagnosed Glioblastoma Multiforme	DC Vaccine	PHASE2	Autologous Dendritic Cell DRUG: Temozolomide PROCEDURE: Radiotherapy	Glioblastoma|Brain Tumor	COMPLETED
NCT02332889	Phase I/II: Decitabine/Vaccine Therapy in Relapsed/Refractory Pediatric High Grade Gliomas/Medulloblastomas/CNS PNETs	DC Vaccine	PHASE1|PHASE2	Vaccine (autologous dendritic cells) DRUG: Decitabine and Hiltonol	Malignant Glioma	TERMINATED
NCT01792505	Dendritic Cell Vaccine With Imiquimod for Patients With Malignant Glioma	DC Vaccine	PHASE1	Dendritic Cell Vaccine in combination with Imiquimod cream	Malignant Glioma Glioblastoma Multiforme Anaplastic Astrocytoma High Grade Glioma	COMPLETED
NCT01808820	Dendritic Cell (DC) Vaccine for Malignant Glioma and Glioblastoma	DC Vaccine	PHASE1	Dendritic Cell Vaccine DRUG: Imiquimod	GBM Glioblastoma Glioma Malignant Glioma	COMPLETED
NCT01635283	Vaccine for Patients With Newly Diagnosed or Recurrent Low-Grade Glioma	DC Vaccine	PHASE2	tumor lysate-pulsed autologous dendritic cell vaccine	Glioblastoma Multiforme	COMPLETED
NCT02049489	A Study of ICT-121 Dendritic Cell Vaccine in Recurrent Glioblastoma	DC Vaccine	PHASE1	ICT-121 DC vaccine	Glioblastoma Glioblastoma Multiforme Glioma Astrocytoma Brain Tumor	COMPLETED
NCT02010606	Phase I Study of a Dendritic Cell Vaccine for Patients With Either Newly Diagnosed or Recurrent Glioblastoma	DC Vaccine	PHASE1	Dendritic cell vaccination in addition to standard temozolomide chemotherapy and involved field radiation therap	Glioblastoma Malignant Glioma Medulloblastoma Recurrent Pediatric Glioblastoma Multiforme Pediatric Brain Tumor, Recurrent Pediatric Brain Tumor	COMPLETED
NCT04552886	Dendritic Cell Vaccination With Standard Postoperative Chemoradiation for the Treatment of Adult Glioblastoma	DC Vaccine	PHASE1	TH-1 Dendritic Cell Immunotherapy	Glioblastoma Multiforme	COMPLETED
NCT00589875	Phase 2a Study of CAN-2409 With Standard Radiation Therapy for Malignant Glioma	Acid Vaccine	PHASE2	CAN-2409 Valacyclovir, Temozolomide Radiation therapy	Malignant Glioma Glioblastoma Multiforme|Anaplastic Astrocytoma,High Grade Glioma	COMPLETED
NCT05685004	Study of Neoantigen-specific Adoptive T Cell Therapy for Newly Diagnosed MGMT Negative Glioblastoma Multiforme (GBM)	Personalized cellular vaccine	PHASE2|PHASE3	TVI-Brain-1 PROCEDURE: Standard of Care Radiotherapy Temozolomide	Glioblastoma Multiforme of Brain	RECRUITING
NCT01290692	Study To Test the Safety and Efficacy of TVI-Brain-1 As A Treatment for Recurrent Grade IV Glioma	Personalized cellular vaccine	PHASE2	TVI-Brain-1	Grade IV Glioma Grade IV Astrocytoma Glioblastoma Multiforme	COMPLETED
NCT02709616	Personalized Cellular Vaccine for Glioblastoma (PERCELLVAC)	Personalized cellular vaccine	PHASE1	Personalized cellular vaccine	Glioblastoma	COMPLETED
NCT02808364	Personalized Cellular Vaccine for Recurrent Glioblastoma (PERCELLVAC2)	Personalized cellular vaccine	PHASE1	Personalized cellular vaccine	Malignant Glioma Glioblastoma Multiforme Anaplastic Astrocytoma High Grade Glioma	COMPLETED
NCT05366062	ERC1671 to Treat Malignant Gliomas When Given in Combination With GM-CSF, Cyclophosphamide, Bevacizumab and Pembrolizumab	Personalized cellular vaccine	PHASE2	ERC1671 GM-CSF, Cyclophosphamide|, Pembrolizumab	Glioma, Malignant	NOT_YET_RECRUITING
NCT03018288	Radiation Therapy Plus Temozolomide and Pembrolizumab With and Without HSPPC-96 in Newly Diagnosed Glioblastoma (GBM)	Personalized cellular vaccine	PHASE2	Pembrolizumab HSPPC-96, Temozolomide Surgery	Glioblastoma	TERMINATED
NCT01814813	Vaccine Therapy With Bevacizumab Versus Bevacizumab Alone in Treating Patients With Recurrent Glioblastoma Multiforme That Can Be Removed by Surgery	Personalized cellular vaccine	PHASE2	HSPPC-96 bevacizumab	Recurrent Glioblastoma Recurrent Adult Brain Tumor Gliosarcoma	TERMINATED
NCT00766753	Vaccination-Dendritic Cells With Peptides for Recurrent Malignant Gliomas	DC Vaccine; Peptide Vaccine	PHASE1|PHASE2	Dendritic vaccine pulsed with multiple peptides	Gliomas Medulloblastoma Neuroectodermal Tumors Primitive	COMPLETED
NCT00846456	Safe Study of Dendritic Cell (DC) Based Therapy Targeting Tumor Stem Cells in Glioblastoma	DC Vaccine; Acid Vaccine	PHASE1|PHASE2	Dendritic cell vaccine with mRNA from tumor stem cells	Recurrent Central Nervous System Neoplasm	COMPLETED
NCT00890032	Vaccine Therapy in Treating Patients Undergoing Surgery for Recurrent Glioblastoma Multiforme	DC Vaccine; Acid Vaccine	PHASE1	BTSC mRNA-loaded DCs	Malignant Neoplasms of Brain	COMPLETED
NCT00626483	Basiliximab in Treating Patients With Newly Diagnosed Glioblastoma Multiforme Undergoing Targeted Immunotherapy and Temozolomide-Caused Lymphopenia	DC Vaccine; Acid Vaccine	PHASE1	RNA-loaded dendritic cell vaccine	Malignant Neoplasms of Brain	COMPLETED
NCT02366728	DC Migration Study for Newly-Diagnosed GBM	DC Vaccine; Acid Vaccine	PHASE2	Unpulsed DCs Human CMV pp65-LAMP mRNA-pulsed autologous DCs	Glioblastoma	COMPLETED
NCT03927222	Immunotherapy Targeted Against Cytomegalovirus in Patients With Newly-Diagnosed WHO Grade IV Unmethylated Glioma	DC Vaccine; Acid Vaccine	PHASE2	Human CMV pp65-LAMP mRNA-pulsed autologous DCs containing GM CSF Temozolomide|BIOLOGICAL: Tetanus-Diphtheria Toxoid (Td) 111-Indium-labeling of Cells for in vivo Trafficking Studies	Glioblastoma	TERMINATED
NCT03688178	DC Migration Study to Evaluate TReg Depletion In GBM Patients With and Without Varlilumab	DC Vaccine; Acid Vaccine	PHASE2	Human CMV pp65-LAMP mRNA-pulsed autologous DCs Temozolomide Varlilumab	Glioblastoma	ACTIVE_NOT_RECRUITING
NCT01326104	Vaccine Immunotherapy for Recurrent Medulloblastoma and Primitive Neuroectodermal Tumor	DC Vaccine; Acid Vaccine	PHASE2	TTRNA-xALT TTRNA-DCs	Medulloblastoma Neuroectodermal Tumor	ACTIVE_NOT_RECRUITING
NCT03615404	Cytomegalovirus (CMV) RNA-Pulsed Dendritic Cells for Pediatric Patients and Young Adults With WHO Grade IV Glioma, Recurrent Malignant Glioma, or Recurrent Medulloblastoma	DC Vaccine; Acid Vaccine	PHASE1	CMV-DCs with GM-CSF	Giant Cell Glioblastoma Recurrent Glioblastoma Recurrent Gliosarcoma	COMPLETED

Among these trials, more than half use peptide/protein vaccines, followed by DC vaccines and nucleic acid vaccines. In terms of progress, the majority of clinical trials are still in Phase I or Phase II, with only a few advancing into Phase III. **Table 1** presents clinical trial information for some of the glioma therapeutic vaccines.

## 6 Challenges and outbreak for the application of tumor vaccines in glioma

The development of therapeutic vaccines against glioma confronts multifaceted biological challenges. A primary challenge stems from the immunosuppressive TME, characterized by cellular components including regulatory T cells (Tregs) and myeloid-derived suppressor cells (MDSCs) that actively suppress anti-tumor immunity (14). Furthermore, the intratumoral heterogeneity of glioma drives clonal diversity in tumor-associated antigen expression, complicating the design of vaccines with broad-spectrum efficacy (14). Tumor-intrinsic immune evasion mechanisms, particularly downregulation of major histocompatibility complex (MHC) molecules, impair the antigen recognition capacity of vaccine-primed cytotoxic T cells (130). The blood-brain barrier (BBB) imposes additional pharmacokinetic constraints, limiting both vaccine component delivery and immune effector infiltration into intracranial tumor site. Strategic focus on epitope spreading and rational antigen selecting may may provide critical breakthroughs for overcoming these biological barriers in glioma immunotherapy.

### 6.1 Blood-brain barrier challenges in glioma vaccine delivery

The BBB is a significant obstacle in delivering therapeutic agents, including vaccines, to the brain. Although peripheral vaccines can activate systemic immunity, they lack sufficient local CNS immune infiltration and fail to address the barrier effects of intratumoral immunosuppressive cells, such as Tregs and MDSCs (131). The failure of the EGFRvIII-targeted vaccine (Rindopepimut) in phase III trials was attributed in part to the inability of vaccine-induced antibodies to penetrate the BBB, coupled with tumor microenvironment-mediated suppression of cytotoxic T cell activity (132). Therefore, the BBB presents a major challenge in the clinical application of glioma vaccines, which require efficient delivery of antigens, adjuvants, and immune cells to activate the immune system within the tumor microenvironment.

Various strategies are being explored to overcome the BBB, such as the use of nanoparticle-based delivery systems, which can encapsulate antigens and adjuvants to facilitate their crossing into the CNS. Lipid nanoparticles (LNPs), in particular, have shown promise in overcoming the BBB and enhancing vaccine efficacy (133). These nanoparticles can encapsulate RNA or DNA vaccines and allow for targeted delivery directly to tumor tissues. In a preclinical model of glioma, LNPs carrying mRNA encoding a tumor antigen successfully crossed the BBB and induced a robust immune response, leading to the reduction of glioma cell proliferation (134). Another approach involves the use of focused ultrasound (FUS) to temporarily open the BBB. FUS, when combined with microbubbles, can induce localized disruption of the BBB, allowing for the delivery of larger molecules like vaccines (135, 136). While these techniques show significant potential, more research is needed to determine their safety and efficacy in human patients, particularly regarding the long-term effects of disrupting the BBB. Numerous vaccine designs have inadequately addressed the challenge of precision delivery to specific brain regions or cell types (137). For instance, although certain LNPs can traverse the BBB, their inability to selectively target distinct brain cell populations results in suboptimal intraparenchymal distribution (138). This limitation may compromise therapeutic efficacy, as certain neurological disorders necessitate precise interventions in defined cellular subtypes. Future research should prioritize the refinement of vaccine formulations and delivery systems to enhance BBB penetration capacity and targeting specificity, thereby improving therapeutic outcomes in glioma management.

### 6.2 Immunosuppressive tumor microenvironment

The immunosuppressive TME in glioma is a key determinant of immunotherapy resistance. Glioma TME exerts immunosuppression through multiple integrated mechanisms, including the infiltration of immunosuppressive cells such as M2-polarized glioma-associated macrophages (GAMs), Tregs and MDSCs; the secretion of immunosuppressive cytokines like transforming growth factor-beta (TGF-β) and interleukin-10 (IL-10); the paucity of TILs, and the upregulated expression of inhibitory immune checkpoint molecules such as PD-1, T cell immunoglobulin and mucin-domain containing-3 (TIM-3), and lymphocyte activation gene-3 (LAG-3) (139–141). In glioma, GAMs are the most abundant cell type and can be functionally divided into pro-inflammatory M1 and immunosuppressive M2 (142). M2-polarized GAMs secrete IL-10 and TGF-β, while low levels of IL-12, creating an immunosuppressive effects in the TME and also promoting the proliferation of glioma stem cells (GSCs) (141, 143). T cell may become dysfunctional after infiltrating the TME through various mechanisms such as replicative senescence, functional exhaustion, and clonal deletion (139). Glioma cells and certain immune cells release multiple immunosuppressive factors into the TME, such as TGF-β and IL-10, which can attract and activate immunosuppressive cells (such as tumor-associated macrophages (TAMs) and Treg cells), while inhibiting the activation of APCs and effector immune cells (144, 145).

Combination ICIs (such as anti-PD-1, PD-L1, and CTLA-4) with tumor vaccines enhances antitumor immunity through IFN-γ-mediated recruitment of peripheral immune effectors into tumor lesions. Curran et al. conducted a study on the melanoma murin model, which found that combining tumor antigen vaccines with PD-1 and CTLA-4 blockade significantly prolonged the survival of mice and increased intratumoral CD8^+^/Treg ratios (146). Molecular stratification of gliomas through integrated multi-omics analysis using machine learning algorithms enables identification of distinct immune microenvironment subtypes correlated with glioma WHO grades, thereby formulating personalized immunotherapeutic strategies. Keskin Et al. treated 8 glioma patients with personalized neoantigen vaccines, and the results showed that 6/8 dexamethasone-free glioma patients developed polyfunctional neoantigen-specific T cell (105). The number of TILs increased, and vaccine-induced T cells could migrate from peripheral blood to the brain, thereby altering the immunological microenvironment of glioma (105).

### 6.3 Immune evasion

The host immune system could eliminate malignant cell precursors and control minimal tumors in a dynamic equilibrium to prevent cancer expansion, until tumor cells acquire genetic or epigenetic alterations that enable them to evade the immune system. This immune evasion phenotype can be classified under a new conceptual framework of “3 C” (147): (1) Camouflage, hiding cancer cells to avoid immune recognition; (2) Coercion, directly or indirectly interfering with immune effector cells; (3) Cellular protection, protecting malignant cells from the cytotoxic effects of immune cells.

Specifically, glioma cells escape immunosurveillance by evading immune cells, avoiding detection or recognition as tumor cells. This camouflage may result from defective antigen presentation, limited chemokine secretion (ICD - related or not), or matrix barriers blocking immune infiltration (148–150). When camouflage fails, tumor cells suppress immune effectors (DCs, NK cells, TH1 - polarized CD4^+^ T cells, CD8^+^ CTLs) and boost immunosuppressive cells (CD4^+^ CD25^+^ FOXP3^+^ Treg cells, specific TAM subsets, MDSCs) (150–152). Such coercive activity may stem from altered immune - modulating ligand expression on cancer cells, defective DAMP/pro - inflammatory cytokine signaling, and/or release of immunomodulatory metabolites in the TME (149, 153, 154). CD8^+^ CTLs and NK cells kill cancer cells via “immunological synapses”, structured connections enabling targeted release of cytotoxic molecules (GZMB, PRF1), expression of death receptor ligands (Fas ligand FASLG), and release of tumor - directed cytokines (especially IFNγ) (155, 156). Understanding these mechanisms has driven revolutionary advances in cancer immunotherapy and the development of various therapeutic strategies, including monoclonal antibodies, proteins, nucleic acids, and immune-active cells, all aimed at activating or enhancing the immune response against tumors.

In tumor immunotherapy, engagement of multi-tiered immune activation pathways provides a strategic approach to counter antigen presentation defects enabling immune escape. For instance, β2-microglobulin (β2M) mutations allow tumor cells to escape T cell-mediated cytotoxicity, thereby compromising multi-epitope vaccines efficacy and epitope spreading. In such scenarios, mobilization of innate immune cells may circumvent antigen loss. Recent research has found that innate lymphoid cells (ILC1 and ILC2) play critical roles in tumor immune surveillance. ILC1s bridge the innate and adaptive immune lineages, and establish a new immune surveillance module independent of traditional T cell-mediated pathway (157). ILC2s, dependent on the GATA-binding protein 3 (GATA3) transcription factor for development, exert anti-tumor activity by secreting type 2 cytokines such as IL-4 and IL-13 in the tumor microenvironment, influencing tumor prognosis (158). Vinod P. Balachandran et al. demonstrated that an engineered IL-33 protein effectively expand ILC2s and tertiary lymphoid structures (TLS). This intervention significantly augmented anti-tumor immune response and demonstrated therapeutic potential in a PDAC mouse model (159). Neutrophils have been shown to contribute substantially to tumor clearance when T cell-targeted immunotherapies are administered. Edgar G. Engleman et al. revealed that CD40 agonistic antibodies could enhance neutrophil cytotoxic activity and induce granulopoiesis. This mechanism promotes antibody-dependent cellular cytotoxicity (ADCC) through Fc gamma receptor (FcγR) signaling, thereby improving tumor eradication (160). Furthermore, the combination of OX40 co-stimulation or CTLA-4 blockade with melanoma-specific CD4^+^ T cell therapy achieves comprehensive tumor elimination, including targeting antigen escape variants (161). Current evidence indicates that complete tumor regression requires neutrophils-mediated cytotoxicity and partially relies on inducible nitric oxide synthase (iNOS) activity. These findings collectively suggest that orchestrating multi-tier immune responses, particularly by harnessing innate immune components (such as ILC1, ILC2, and neutrophils), can effectively overcome immune escape caused by defective antigen presentation, providing novel strategic approaches for tumor immunotherapy.

### 6.4 Tumor heterogeneity

Tumor heterogeneity poses a major challenge for glioma vaccine therapeutics. This biological phenomenon manifests as significant molecular and genetic diversity within tumors, resulting in intercellular variations in proliferation rates, invasive potential, and therapeutic susceptibility (14). In a Phase III non-randomized controlled trial involving glioma patients, DCVax-L treatment demonstrated a significant extension of mOS compared to standard care, highlighting the critical influence of tumor heterogeneity on the vaccine efficacy (25). Tumor heterogeneity drives two key limitations of monovalent vaccine approaches: (1) treatment-induced antigen loss that facilitates immune escape, and (2) inadequate coverage of patient-specific tumor antigens. These limitations underscore that vaccine designs must address tumor molecular diversity and the induction of polyclonal T cell against multiple neoantigens to prevent the clinical ineffectiveness of single-target vaccines caused by tumor heterogeneity. Researchers at the University of Florida have pioneered an innovative mRNA vaccine platform utilizing autologous tumor-derived mRNA complexes. This approach enhances dendritic cell antigen presentation through optimized mRNA clusters loading, enabling rapid immune priming that generates potent tumor-specific cytotoxicity (129).

### 6.5 Inducing epitope spreading for anti-tumor vaccine efficacy

Epitope spreading is an immunological phenomenon in which the immune system initially responds to pathogen’s dominant epitopes and subsequently generates responses to additional epitopes (including hidden epitopes) over time, ultimately leading to more extensive immune responses (162). In cancer immunotherapy, inducing epitope spreading is highly desirable, as it promotes a more comprehensive and sustained immune response against tumor cells. This approach helps combat tumor heterogeneity and mitigates the risk of immune escape by targeting multiple tumor-associated antigens. Clinically, patients demonstrating epitope spreading typically exhibit sustained anti-tumor immunity, which correlates with improved clinical outcomes (163).

**Figure 3:**
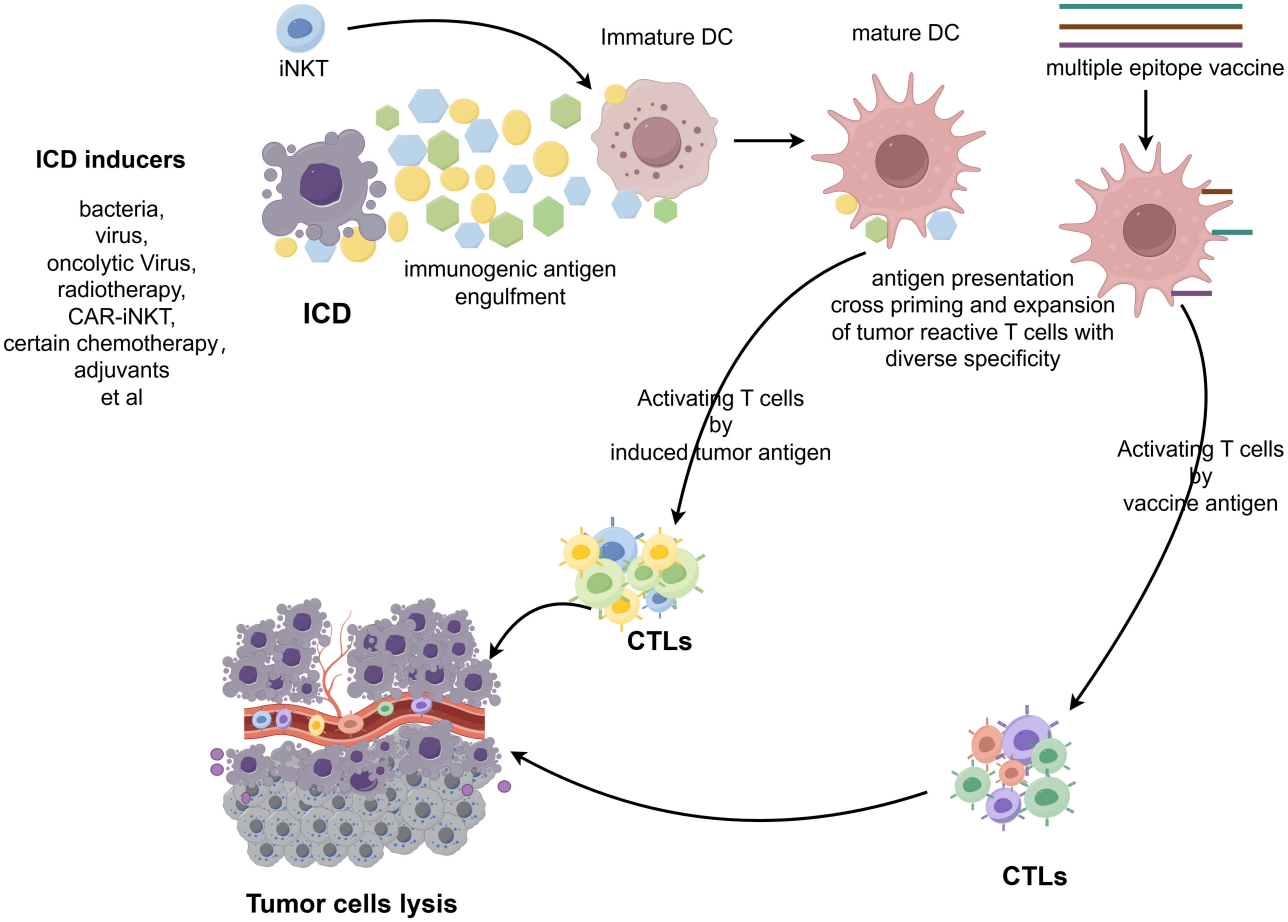
Schematic illustration of the process of inducing epitope spreading for anti-tumor vaccine efficacy. The figure was generated by Figdraw (https://www.figdraw.com).

A critical mechanism for promoting epitope spreading is tumor immunogenic cell death (ICD), which is a programmed cell death that actively engages both innate and adaptive immunity. Unlike non-immunogenic apoptosis, ICD occurs only when three key conditions are met: antigenicity (exposure of TSAs), adjuvanticity (release of danger signals), and a permissive tumor microenvironment. ICD can be triggered by diverse modalities, including: radiotherapy, specific chemotherapy agents (e.g., doxorubicin, oxaliplatin, cyclophosphamide), immune adjuvants, and microbial agents (e.g., bacterial components, oncolytic viruses) (164). The immunogenic effects of ICD are mediated via three key mechanisms: (1) Release of Damage-Associated Molecular Patterns (DAMPs) – These molecules serve as danger signals, activating DCs and other APCs. (2) Exposure of tumor-associated antigens – Dying tumor cells release neoantigens, expanding the repertoire of immune-targetable epitopes. (3) Cross-priming of T cells – APCs phagocytose tumor antigens and present them to naïve T cells, thereby broadening the immune response through epitope spreading. By generating an *in situ* vaccination effect, ICD amplifies epitope spreading, enabling T cells to target heterogeneous tumor subclones and diminishing the likelihood of immune escape. This synergistic interaction between ICD and immunotherapy represents a promising therapeutic strategy (165) (**Figure 3**).

Strategies that induce epitope spreading, particularly through ICD, play a critical role in cancer therapy. By broadening immune recognition breadth and sustaining antigen-specific responses, these approaches counteract tumor heterogeneity and immune evasion, establishing more durable anti-tumor immunity.

#### 6.5.1 Induction of epitope spreading combined with radiotherapy or chemotherapy

Combining tumor vaccines with radiotherapy or specific chemotherapy regimens enhance the release of immunogenic tumor antigens due to tumor cell destruction, potentially promoting epitope spreading. For instance, low-dose radiotherapy can amplify antigen presentation by inducing ICD, thereby broadening antitumor immune responses. In a Phase Ib clinical trial of the personalized neoantigen vaccine NEO-PV-01 combined with first-line therapy for advanced non-squamous non-small cell lung cancer (NSCLC), 69% of patients (9/13) not only exhibited neoantigen-specific CD4^+^ and CD8^+^ T cell immune responses but also elicited epitope spreading toward non-vaccine neoantigens, including responses targeting KRAS G12C and G12V mutations (166). Longitudinal evaluation of epitope spreading in 3 patients at 52 weeks post-vaccination revealed that 80% of epitope-specific responses persisted at later time points, demonstrating the long-term persistence of these immune response.

#### 6.5.2 Oncolytic viruses inducing epitope spreading

Oncolytic viruses (OVs) are genetically engineered to selectively infect tumor cells and induce ICD, triggering the release of numerous tumor antigens. These antigens are subsequently processed and presented by DCs, priming a polyclonal immune response that targets both the vaccine-directed epitopes and additional tumor-related neoantigens through epitope spreading synergistically enhances immune activation, as the newly released neoantigens provide additional targets for vaccine-primed lymphocytes (167, 168). In glioma therapeutics, OV vaccines have shown significant therapeutic potential. A 2021 study utilizing a modified HSV-1 virus for the treatment of pediatric high-grade gliomas achieved a mOS of 12.2 months, significantly surpassing the 5.6-month mOS observed in historical controls (169). A groundbreaking 2024 *Nature Cancer* study further advanced this paradigm by engineering an OV encoding bystander T cell epitopes (BYTE) (170). In human melanoma mouse models, OV-BYTE therapy initially targeted viral epitopes but subsequently induced epitope spreading that enhanced tumor-specific T cell responses, leading to more robust and widespread anti-tumor immunity.

#### 6.5.3 Neoantigen vaccines and adjuvant synergy induced epitope spreading

Neoantigen vaccines, a class of highly personalized therapeutics, target tumor-specific mutations by eliciting robust immune responses. Intriguingly, this process may enhance immune recognition of non-vaccine tumor antigens through DC-mediated cross-presentation following T cell activation, leading to epitope spreading. A Phase I trial demonstrated that all 8 patients with advanced melanoma who underwent surgery and received NeoVax treatment (with polyICLC as an adjuvant) achieved 4-year overall survival, with 6 maintaining disease-free status (171). Additionally, two patients exhibited CD4^+^ T cell reactivity against non-vaccine neoantigens, accompanied by tumor-infiltrating neoantigen-specific T cell clonal. This provides direct evidence of vaccine-induced epitope spreading and subsequent tumor cell elimination. Nevertheless, as active immunotherapies, such vaccines face challenges in overcoming the immunosuppressive tumor microenvironment, resulting in suboptimal efficacy of current shared neoantigen vaccines.

To address this challenge, integrating more potent adjuvants into tumor vaccines represents a promising strategy to enhance DC-mediated antigen presentation, amplify T cell activation, and recruit diverse immune cellpopulations. This approach facilitates broader tumor antigen recognition through epitope spreading mechanisms. Established adjuvants known to induce epitope spreading include polyICLC and Montanide ISA 51 (172–174). Mørk et al. recently demonstrated the clinical potential of this strategy by treating five patients with unresectable, ICI-refractory metastatic melanoma using personalized neoantigen peptide vaccines combined with the novel adjuvant CAF^®^09b (175). Their findings revealed that one patient developed durable neoantigen-specific immune responses with T cells demonstrating cross-reactivity to multiple neoantigens, suggesting this approach may effectively overcome tumor immune evasion mechanisms.

#### 6.5.4 Vaccine combined with cell therapy to induce antigen spreading

While CAR-T cell therapy inherently exhibits limited capacity to induce epitope spreading, emerging evidence suggests that vaccine co-administration can augment CAR-T cell metabolic fitness and polyfunctionality. This combinatorial approach enhances bidirectional interactions between CAR-T cells and the endogenous immune network, thereby facilitating epitope spreading (176). Such immunological diversification enables recognition of a broader tumor antigen repertoire, including neoantigens, ultimately enhancing therapeutic efficacy. The progressive elimination of malignant cells through this mechanism establishes a self-perpetuating cycle of amplified immune activation correlated with improved clinical outcomes. A recent study published in *Cell* found that neither monotherapy with CAR-T cells nor vaccines effectively induced antigen spreading. However, synergistic administration of amphiphile vaccine (amph-vax) with CAR-T cell therapy (CAR T-vax) elicited robust CD8^+^ T cell responses specific to the ovalbumin-derived SIINFEKL epitope while concurrently activating host T cell immunity against non-Trp1 tumor antigens (176). These findings indicate that vaccine-potentiated CAR-T therapy promotes antigen spreading, mobilizing endogenous T cell responses against secondary tumor antigens even in neoantigen-deficient microenvironments.

Invariant natural killer T (iNKT) cells exhibit intrinsic adjuvant properties through CD40L expression and IFN-γ secretion upon activation (177, 178). These effector functions directly promote DCs maturation and functional licensing, establishing an immunological foundation conducive to epitope spreading and systemic immune potentiation (179). Zhou Xin et al. have recently demonstrated that CAR- iNKT cells not only mediate direct tumor cytotoxicity but also orchestrate endogenous CD8^+^ T cell responses against secondary tumor antigens, including ovalbumin (OVA)-derived epitopes, in B16-hCD19 murine models (180). This study mechanistically validated CAR-iNKT cells’ capacity to induce epitope spreading in solid tumor, further highlighting their translational potential as combinatorial partners with vaccine-based immunotherapies.

### 6.6 Antigen selection bottleneck

**Figure 4:**
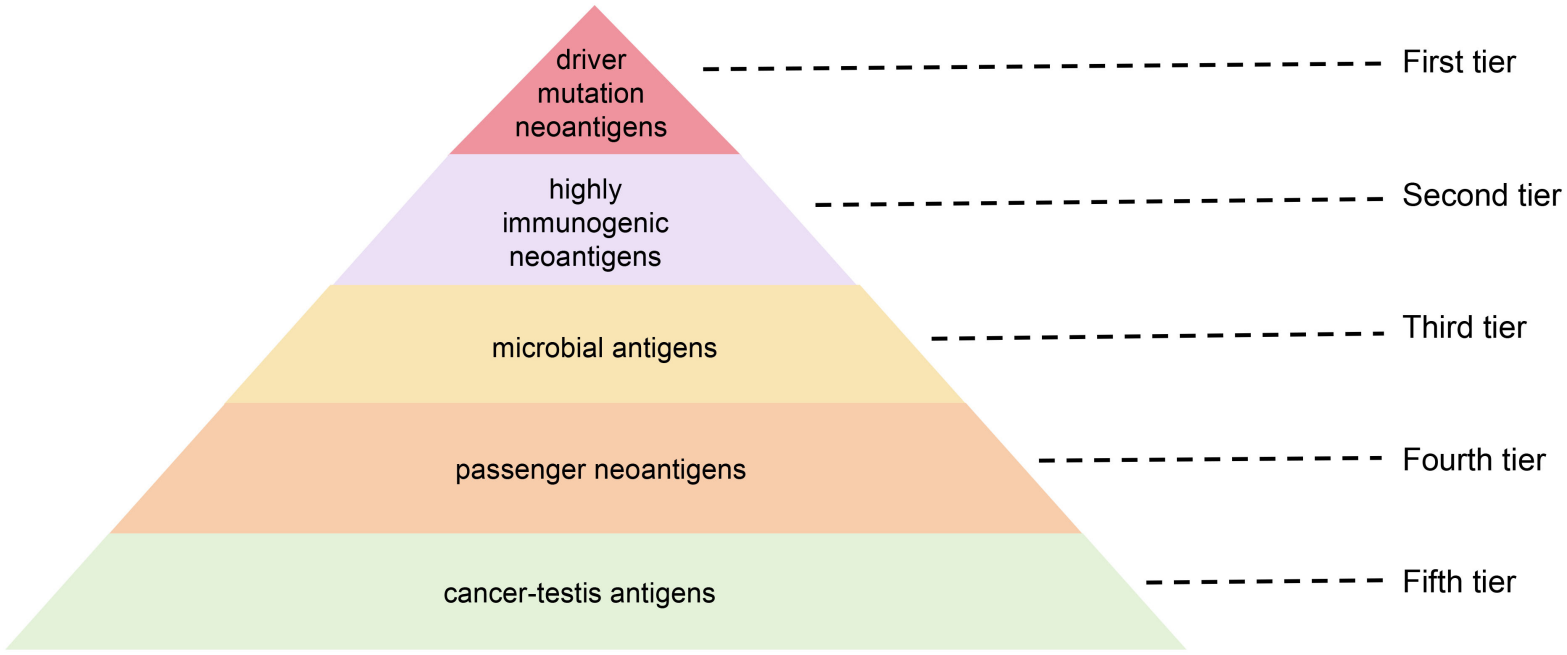
Stratified prioritization of antigenic targets for glioma vaccines: a five-tier hierarchy based on immunogenicity and functional relevance.

The efficacy of tumor vaccines depends on antigen quality and immunogenicity. Therefore, strategic antigen selection is crucial to improve the quality of the selected tumor antigens and prioritize them. The tiered antigen ranking system, typically categorizing targets based on tumor specificity, therapeutic relevance, and immune recognition potential, constitutes a cornerstone in rational vaccine design and clinical translation. (**Figure 4**).

#### 6.6.1 First tier: neoantigens from driver mutations

Clones harboring driver mutations are generally conserved across tumor evolution. Driver genes play a critical role in sustaining malignant phenotype—including proliferation, invasion, and metastasis— and exhibit consistent expression patterns in both primary and metastatic lesions. Consequently, these antigens retain potent immunogenicity unaffected by central immune tolerance mechanisms, positioning them as ideal targets for cancer vaccines. This conservation enables them to circumvent therapeutic resistance arising from tumor heterogeneity, thereby reducing the likelihood of immune escape (181). Furthermore, shared neoantigens derived from driver mutations are tumor-specific, bypassing thymic negative selection. Consequently, these antigens retain potent immunogenicity unaffected by central immune tolerance mechanisms, positioning them as ideal targets for cancer vaccines (182). TG01, a peptide vaccine targeting seven high-frequency KRAS G12/G13 codon mutations, has entered clinical trials (183). The results demonstrated that favorable safety in the low-dose group cohort while eliciting robust T cell responses across all dose groups. Survival analyses revealed 2-year and 3-year survival rates of 72% and 37%, respectively, underscoring the translational promise of conserved neoantigen vaccines.

#### 6.6.2 Second tier: neoantigens with higher exogeneity

Exogeneity refers to the degree of foreignness of neoantigens compared to their wild-type protein. Neoantigens exhibiting higher exogeneity typically demonstrate minimal homology to native cellular proteins. Notably, certain mutation-derived neoantigens and CSEs may possess elevated foreignness levels. Immunological studies establish an inverse relationship between endogenous neoantigen similarity and immunogenicity: neoantigens closely resembling self-proteins exhibit attenuated immunogenicity and increased susceptibility to immune tolerance-mediated clearance (184). Conversely, neoantigens with higher exogeneity display enhanced immunogenic potential, enabling their recognition as non-self entities by immune effectors. This property allows them to evade central tolerance mechanisms while priming antitumor immune responses (62). mRNA-4157, a lipid nanoparticle-formulated RNA neoantigen vaccine, demonstrated tumor-specific T cell activation in early-phase clinical testing (185). These studies underscore the therapeutic promise of targeting neoantigens with pronounced structural foreignness in cancer immunotherapy.

#### 6.6.3 Third tier: microbial antigens

Microbial antigens exhibit distinct structural recognized by the immune system as non-self entities, conferring inherent tumor-targeting specificity. When administered as vaccine components, these antigens can elicit the specific immune responses, including the activation of T cells and B cells (114). This specific immune response can target tumor cells expressing similar antigens, as certain tumors abnormally express pathogen-associated antigens or display antigenic structures resembling those of pathogens. These shared epitopes provide critical targets for tumor vaccine development (186). In a Phase I clinical trial of the pp65-DC vaccine for glioma treatment, published in April 2017, the median PFS and OS were significantly extended to 25.3 months (vs. 8.0 months with standard therapy) and 41.1 months (vs. 19.2 months with standard therapy), in 11 enrolled glioma patients (187). Notably, four patients receiving ≥3 postoperative vaccine dose remained progression-free for 59–64 months. A 2021 study published in *Nature (* 115) identified abundant bacterial antigens in melanoma cells. These bacterial peptides are presented on tumor cell surfaces through HLA complexes, enabling T cells recognition and immune responses. Serving as potential novel tumor antigens, these microbial epitopes represent promising targets for vaccine development to enhance antitumor immunity.

#### 6.6.4 Fourth tier: passenger mutation neoantigens

Passenger neoantigens are tumor-specific epitopes arising from “passenger” mutations that lack oncogenic functions but can generate immunogenic neoepitopes. Unlike driver mutations critical for tumorigenesis, these passenger mutations may produce HLA-presented epitopes recognized by T cell receptors, thereby providing targets for immunotherapeutic strategies (188). Clinical translation of this concept is exemplified by GEN-009, a personalized vaccine comprising 4–20 synthetic long peptides selected via the ATLAS immunogenicity prediction platform adjuvanted with poly-ICLC. In the multicenter Phase I/IIa trial (NCT03633110), this vaccine demonstrated favorable safety profiles in 8 high-risk solid tumors patients, eliciting antigen-specific CD4^+^/CD8^+^ T cell responses against ≥1 neoantigen per patient. Longitudinal analysis revealed evidence of epitope spreading, suggesting broader immune activation. (ASCO 2021, Abstract 2539).

#### 6.6.5 Fifth tier: cancer-testis antigens

CTAs originate from developmental genes epigenetically silenced in somatic tissues through DNA hypermethylation, with physiological expression restricted to germline and placental cells. Tumor-specific CTA re-expression occurs via epigenetic dysregulation, generating immunogenic peptides presented via MHC molecules. CTAs can circumvent central immune tolerance and elicit antigen-specific T cell responses, representing promising immunotherapy targets (189). The X chromosome-encoded MAGE-A protein family demonstrates oncogenic expression in across multiple malignancies. In a Phase II trial, postoperative MAGE-A3^+^ NSCLC patients (n=182) receiving MAGE-A3 antigen-specific cancer immunotherapy (ASCI) exhibited significantly reduced recurrence rates (30.6% vs 43.3% placebo) at primary analysis (190). However, clinical development of MAGE-A3-targeted therapies encountered unexpected toxicity (191, 192). These adverse events likely stem from T cell receptor cross-reactivity with structurally homologous epitopes expressed in normal tissues, underscoring the critical importance of achieving complete target exclusivity in cancer immunotherapy.

### 6.7 Optimizing delivery systems to enhance vaccine efficacy

The delivery system is crucial for the immune efficacy of vaccines. In novel vaccine research, nanoparticles, viral vectors, or cell membrane-coated vaccines are utilized to achieve delivery, ensuring specific enrichment of neoantigens around tumor cells (193). Darrell J. Irvine et al. developed lymph node-targeted molecular vaccines by designing an albumin-hitchhiking delivery approach (194). Experimental results demonstrated that amphiphilic molecules injected into mice effectively accumulated in lymph nodes and exhibited superior immune efficacy compared to unmodified molecules. One endogenous LNP formulation, 113-O12B, demonstrated lymph nodes-targeting capabilities. When encapsulating mRNA, it enhanced CD8^+^ T cell responses against the full-length ovalbumin (OVA) model antigen. In the OVA-expressing B16F10 melanoma model, this OVA mRNA vaccine also showed improved efficacy. Viral vector vaccines incorporate tumor antigens into viral particles, mimicking viral infections to activate antigen-presenting cells. This mechanism facilitates better recognition and presentation of tumor-associated antigens, promotes CD8^+^ T cell activation, and may induce epitope spreading.

## 7 Conclusion

Glioma remains one of the most challenging malignancies to treat due to their complex molecular landscape, high heterogeneity, and immunosuppressive microenvironment. However, advances in understanding tumor immunology, antigen identification, and immune evasion mechanisms have driven significant progress in glioma immunotherapy, particularly in the development of tumor vaccines. In recent years, glioma treatment using tumor vaccines has advanced markedly, with several vaccine types — including peptide vaccines, DC vaccines, and nucleic acid vaccines — progressing to clinical trials (25, 29, 195). Personalized neoantigen vaccines, which target mutations unique to a patient’s tumor, could revolutionize glioma therapy by enabling highly specific treatments. Combining these vaccines with ICIs, oncolytic viruses, targeted therapies, and standard modalities such as radiotherapy and chemotherapy represents a promising multimodal strategy to enhance clinical outcomes. Nevertheless, significant challenges persist, including overcoming the immunosuppressive TME, addressing antigen heterogeneity and immune escape mechanisms, and resolving the inherent difficulties in delivering vaccines across the BBB (14, 196). Continued research in these areas is crucial for improving the efficacy of glioma vaccines. Future directions in glioma vaccine therapy will likely focus on optimizing vaccine delivery systems, developing synergistic combination therapies to amplify immune responses, and inducing epitope spreading to broaden antigenic targeting (25, 171, 197). Advances in personalized vaccine development, including incorporating neoantigens, will provide more precise and effective therapies, potentially improving patient survival rates and quality of life.

While glioma vaccines remain in early developmental stages, ongoing clinical trials and technological advancements offer hope for novel therapeutic avenues. The integration of immunotherapy into standard glioma treatment regimens, particularly through synergistic combination therapies, is poised to become a cornerstone in managing this aggressive disease.
